# Chromatographic Fingerprint and the Simultaneous Determination of Five Bioactive Components of *Geranium carolinianum* L. Water Extract by High Performance Liquid Chromatography

**DOI:** 10.3390/ijms12128740

**Published:** 2011-12-02

**Authors:** Qiu-Yue Wu, Yang Zhou, Xin Jin, Yue Guan, Min Xu, Li-Fang Liu

**Affiliations:** State Key Laboratory of Natural Medicines, China Pharmaceutical University, Nanjing, Jiangsu 210009, China; E-Mails: qiuyue86811@163.com (Q.-Y.W.); zhouyang871214@126.com (Y.Z.); jinxin8516@126.com (X.J.); guanyue.1989@163.com (Y.G.); daxue302503165@126.com (M.X.)

**Keywords:** *Geranium carolinianum* L. water extract, HPLC fingerprint, quality evaluation, simultaneous determination

## Abstract

A simple and sensitive HPLC method has been developed in combination with fingerprint analysis and simultaneous determination of five markers, namely gallic acid, corilagin, methyl brevifolincarboxylate, ellagic acid and rutin for evaluation and quality control of *Geranium carolinianum* L. water extract. Extraction methods were optimized by comparing the hydrolysis efficiency of geraniin, a major tannin of the herb, resulting in the method of extraction with water under reflux. Water extracts were analyzed by HPLC, with a mobile phase of 0.1% aqueous phosphoric acid (v/v) and acetonitrile in a gradient program within 65 min. Compounds were detected at 274 nm UV wavelength. For fingerprint analysis, 17 peaks were selected as the characteristic peaks to evaluate the similarities of different samples collected from the suburb of Nanjing. The correlation coefficients of similarity were greater than 0.993. In quantitative analysis, the five selected markers showed good regression (*R* > 0.9991) within test ranges, and the average recoveries were between 97.2–101.7% and their RSD values were less than 4.50%. The total contents of the five markers varied from 44.28 to 71.84 mg/g. The method can be very useful for further development of *G. carolinianum* L. extracts and preparations.

## 1. Introduction

*Geranium carolinianum* L. (Geraniaceae) is a commonly used traditional Chinese medicine (TCM) with efficacy of eliminating wind-damp and treating diarrhea. It is clinically used to treat the arthralgia due to wind-dampness, anaesthetization and muscular constriction, bone and muscle ache, and diarrhea. Its water extract has been applied in a formulated well-known preparation, namely *Unguentum Geranii* with demonstrated efficacy in eliminating eczema [1]. It has been reported that *G. carolinianum* L. as well as most of the congeneric plants contain significant amounts of tannins, flavonoids, organic acids, and volatile oils, contributing to the therapeutic effects of this medicinal herb. Pharmacological studies have shown that these components have anti-inflammatory, antitumor, antioxidant, antiviral and antimicrobial activities [2–8]. It has been reported that *G. carolinianum* L. water extract containing 5–40% of corilagin in quantity is effective for the treatment of gastroxia [9].

Very few reports on the quality control of *G. carolinianum* L. have been published so far. In this work, we focused on establishing an effective method to evaluate the quality of this herb for its safe use in clinical practice. Most methods for quality control of medicinal herbs only analyze a few chemical constituents, which is insufficient as they do not reveal all the components present in the chromatographic profile. Recently, HPLC fingerprinting coupled with quantitative determination have been developed and validated for quality control of herbal drugs and their preparations [10–14]. Combination of chemical fingerprint and quantification of multi-ingredients can not only give an overview of all the constituents in TCM, but also quantitate active components. Thus, this approach can be applied to control the quality of TCM effectively.

In the present study, fingerprint and quantitative analysis by HPLC were performed for the characteristic evaluation of *G. carolinianum* L. Samples were extracted with water under reflux as decoction is the common administration form of this herb. An HPLC fingerprint consisting of 17 common peaks was obtained for the first time. Among these common peaks, five target components namely gallic acid, corilagin, methyl brevifolincarboxylate, ellagic acid and rutin, which were the major chemical constituents in the fingerprint with known biological activities, were selected for simultaneous quantification. The newly established method was utilized to analyze 10 samples of *G. carolinianum* L. collected from the suburb of Nanjing, China.

## 2. Results and Discussion

### 2.1. Optimization of Sample Preparation

Genariin was reported to be a major tannin in many species of *Geranium* including *G. carolinianum* L. [15]. Geraniin can be hydrolyze into simple components such as corilagin and brevifolin carboxylic acid in water at high temperature [16]. Thus, the extraction conditions were optimized to achieve the most thoroughly hydrolization of geraniin and obtain a comparative stabile component group which composed the chemical fingerprint of aqueous extracts from this herb.

**Figure 1:**
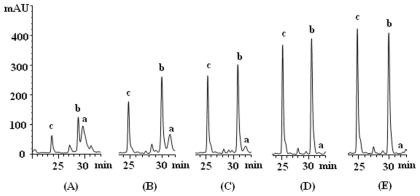
Different hydrolytic degree of “a” into “b” and “c” (proved to be corilagin and brevifolin carboxylic acid, respectively) with different extraction conditions: (**A**) Reflux with 50% methanol for 30 min; (**B**) Reflux with 10% methanol for 30 min; (**C**) Reflux with water for 30 min; (**D**) Reflux with water for 60 min; (**E**) Reflux with water for 90 min.

**Figure 2:**
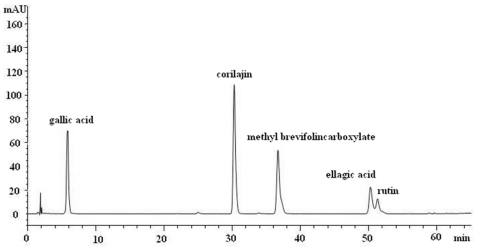
Chromatogram of mixed standard compounds.

As shown in Figure 1, component “a” was identified as geraniin according to published data [15]. Component “b” was identified as corilagin by comparison with the corresponding chemical references under the same condition (Figure 2) and by spiking authentic compounds. Component “c” was identified to be brevifolin carboxylic acid based on its same retention time as that of the hydrolysis product from methyl brevifolincarboxylate. Different extraction conditions including reflux with 50%, 30% and 10% methanol for 30 min and reflux with water for 30, 60, 90 and 120 min were compared. The representative chromatograms shown in Figure 1 revealed that reflux with water for 90 min completely hydrolyzed geraniin into corilagin and brevifolin carboxylic acid. In addition, the five determined components were extracted more efficiently under this condition. Furthermore, the times of extraction were compared and two extraction processes were sufficient to extract the sample. Thus, samples were prepared by reflux with water (90 min × 2) as described in previous section.

### 2.2. Optimization of HPLC Conditions

HPLC conditions were optimized to obtain the desired fingerprints. The combination of acetonitrile and 0.1% aqueous phosphoric acid (v/v) proved to be an optimal mobile phase system for the purpose of chromatographic separation. Final gradient elution method was determined by testing different gradient elution methods. The chromatograms were obtained at a UV wavelength of 274 nm as all tested components have major absorption at this wavelength. Column temperature was kept at 25 °C and the flow rate was set at 1.0 mL/min for optimal separation.

### 2.3. Validation of Fingerprint Analysis

The proposed method for fingerprint analysis was validated in terms of precision, repeatability, and stability. The validation was performed with sample 2 of *G. carolinianum* L. based on the relative retention times (RRTs) and relative peak areas (RPAs). The precision was assessed by analyzing five replicate samples and the relative standard deviation (RSD) was below 2.02% and 4.47% for RRTs and RPAs, respectively. The repeatability and stability (RSD) was below 2.71% and 3.12% (*n* = 5) for RRTs and below 4.50% and 4.90% for RPAs.

### 2.4. Validation of Quantitative Analysis

Standard stock solutions of gallic acid, corilagin, methyl brevifolincarboxylate, and rutin were prepared in 50% (v/v) methanol and of ellagic acid in dimethyl sulfoxide (DMSO) at final concentrations of 0.14, 1.30, 0.10, 0.20, and 0.10 mg/mL, respectively. Calibration was performed by analyzing the five reference solutions in duplicate at six concentration levels, and then the calibration curves were constructed by plotting the peak areas versus the injection quantity of each compound. The limit of detection (LOD) and limit of quantification (LOQ) under the present chromatographic conditions were evaluated at S/N of 3 and 10, respectively. Regression data, LODs, and LOQs for five standard substances are given in Table 1.

The precision was performed by six replicate determinations of five standard solutions. The repeatability was examined by five replications of a sample. To evaluate the stability, the sample solution was injected at 0, 4, 8, 12 and 24 h after preparation. In the recovery test, samples were prepared at three concentration levels in triplicate by spiking known quantities of each of the five standards into the *G. carolinianum* L. sample, and then extracted and analyzed according to the described procedures. The validation data are shown in Table 2.

The robustness was investigated by analysis of the sample solution (sample 2) using two different chromatographic columns, *i.e.*, Agilent Tc-C_18_ column (250 mm × 4.6 mm i.d., 5 μm) and Diamonsil C_18_ column (150 mm × 4.6 mm i.d., 5 μm), and by changing the chromatographic temperature (30 °C instead of 25 °C), the flow rate (1.2 mL/min instead of 1.0 mL/min) or the detection wavelength (280 nm instead of 274 nm), the results (data not shown) indicated that these alterations had little influence on the chromatographic separation and therefore the robustness of the proposed method was fine.

### 2.5. HPLC Fingerprint Analysis

**Figure 3:**
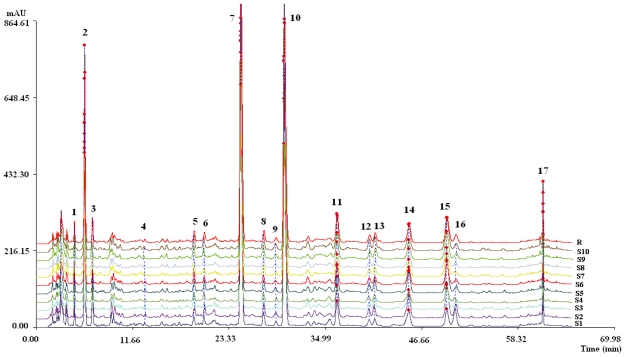
Chromatographic fingerprints obtained from ten batches of *G. carolinianum* L. and the reference fingerprint (mark R) developed with the median of all chromatograms (peaks 1–17 corresponded to 17 common peaks).

Chromatographic fingerprints obtained from ten batches of *G. carolinianum* L. and the reference fingerprint (mark R) developed with the median of all chromatograms are given in Figure 3 and the similarity values of all imported chromatograms with respect to the reference fingerprint are tabulated in Table 3. There were 17 common peaks in all ten batches and the area sum of them accounted for above 90% of the overall peak area. Peaks 2, 10, 11, 15, and 16 were identified as gallic acid, corilagin, methyl brevifolincarboxylate, ellagic acid, and rutin by comparing with the corresponding chemical references under the same condition (Figure 2). Peak 7 was identified to be brevifolin carboxylic acid as mentioned before. The RSDs of RRT and RPA of the other common peaks with respect to peak 10 from all samples were in the ranges of 0.10–3.27% and 6.62–67.24%, respectively, indicating that chemical constituents of *G. carolinianum* L. from different batches were generally consistent with distinct relative peak intensities.

### 2.6. HPLC Quantitative Analysis

The optimal conditions were applied for the quantitative analysis of gallic acid, corilagin, methyl brevifolincarboxylate, ellagic acid, and rutin. Each sample was analyzed in duplicate to determine the mean contents (mg/g) of five selected constituents and the results are shown in Table 4. Corilagin was found to be predominant among the five determined analytes. Many studies have shown that corilagin exhibits anti-tumour, anti-inflammatory and antioxidant activities [17–19]. The high yield of corilagin in water extract may contribute to the curative effect of *G. carolinianum* L. decoction.

## 3. Experimental Section

### 3.1. Materials and Reagents

Ten batches of raw herbs of *G. carolinianum* L. were collected from the suburb of Nanjing, China and listed in Table 3. All the voucher specimens, authenticated by Li-Fang Liu, were deposited at State Key Laboratory of Natural Medicines, China Pharmaceutical University.

**Figure 4:**
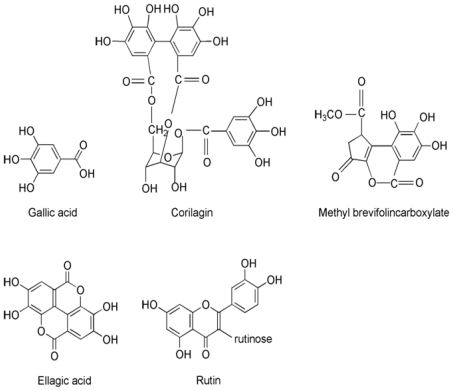
Chemical structures of the five determined analytes.

Acetonitrile of HPLC grade was from Merck (Darmstadt, Germany); distilled water and HPLC grade methanol were used in the analysis. Other reagents were of analytical grade. Gallic acid (Lot: 110831–200302), corilagin (Lot: 111623–200302) and rutin (Lot: 760706) were purchased from the National Institute for the Control of Pharmaceutical and Biological Products. Methyl brevifolincarboxylate and ellagic acid (purity > 98%) were isolated in our laboratory, and their purity and structures were confirmed by HPLC and by comparison of spectral data to those published in the literature. Structures of the standards are illustrated in Figure 4.

### 3.2. Instrumentation and Chromatographic Conditions

All analysis was performed using an Agilent 1200 series HPLC system equipped with a quaternary pump, a vacuum degasser, and data analysis was carried out by Agilent ChemStation Software [20]. The separation was carried out on a Diamonsil C_18_ (2) column (150 mm × 4.6 mm i.d., 5 μm) under the following chromatographic conditions: sample injection volume, 20 μL; column temperature, 25 °C; flow rate, 1.0 mL/min; mobile phase, acetonitrile and 0.1% aqueous phosphoric acid (v/v). A gradient program was used according to the following profile: 0–20 min, 2–10% acetonitrile; 20–45 min, 10–14% acetonitrile; 45–55 min, 14–17% acetonitrile; 55–60 min, 17–30%; 60–65 min, 30–50% acetonitrile. The wavelength of UV detection was set at 274 nm. The chromatogram of mixed standard compounds is shown in Figure 2.

### 3.3. Sample Preparation

The fresh aerial part of *G. carolinianum* L. was dried and pulverized (40 mesh). The aqueous extract was prepared by reflux of 0.5 g of powdered sample with 25 mL water. The reflux was continued for 90 min and repeated once. The extracted solution was combined and filtered and diluted to volume with water in a 50 mL volumetric flask. The solution was filtered through a 0.45 μm millipore membrane prior to HPLC analysis.

### 3.4. Analysis of Chromatographic Fingerprints

“Similarity Evaluation System for Chromatographic Fingerprint of Traditional Chinese Medicine (version 2004A)” [21] recommended by the State Pharmacopoeia Commission of China was introduced to generate reference fingerprint developed with the mean or median of all chromatograms and to calculate the correlative coefficients between different chromatograms and the reference fingerprint [22]. In this study, the peak of corilagin was assigned as a reference to calculate RRT and RPA of each characteristic peak as it was situated in the middle of the chromatogram and showed a large peak area among all the peaks.

## 4. Conclusions

Our current study provides a new HPLC-based method for quality control of *G. carolinianum* L. The representative chemical fingerprint combined with simultaneous determination of five target components offered a powerful and rational way to guarantee the quality of this herb. It is important to highlight that we investigated the hydrolization of geraniin for sample preparation and the results revealed that extraction with water under reflux yielded more stable components including corilagin that has many significant bioactivities. These results support of the rationality of the use of this herbal medicine as a decoction. Furthermore, the reported study may serve as an important reference to establish the quality control method for the extracts and preparations of this herb as well as other related TCM.

## Figures and Tables

**Table 1:** Regression data, limit of detections (LODs) and limit of quantifications (LOQs) for five standard substances.

Standard Substance	Calibration Equation *y* = a*x* + b (a)	*R*(b)	Linear Range (μg/mL)	LOD (μg/mL)	LOQ (μg/mL)
Gallic acid	*y* = 2619.1*x* – 15.114	0.9998	7.00–140.00	0.01	0.035
Corilagin	*y* = 1338.5*x* – 66.433	1.0000	65.00–780.00	0.055	0.22
Methyl brevifolincarboxylate	*y* = 4955.2*x* + 65.426	0.9993	5.00–40.00	0.35	1.00
Ellagic acid	*y* = 2467.0*x* – 3.9683	0.9998	10.00–100.00	0.10	0.25
Rutin	*y* = 961.98*x* – 56.558	0.9991	10.00–160.00	0.70	2.00

(a) *y* and *x* stand for the peak area and the injection quantity (μg) of each standard substance, respectively;

(b) *R* = correlation coefficient, *n* = 6.

**Table 2:** Precision, repeatability, stability and recovery of five standard substances.

Standard Substance	Precision RSD (%) (*n* = 6)	Repeatability RSD (%) (*n* = 5)	Stability RSD (%) (*n* = 5)	Recovery (%) (a) (*n* = 9) Mean RSD (%)	
Gallic acid	1.88	4.44	0.82	97.2	3.19
Corilagin	3.25	0.63	0.54	99.5	3.77
Methyl brevifolincarboxylate	4.99	4.36	1.74	98.0	4.50
Ellagic acid	2.80	4.39	3.28	100.3	4.42
Rutin	2.36	1.67	4.35	101.7	4.21

(a) Recovery (%) = 100 × (amount found-original amount)/amount spiked.

**Table 3:** Raw material samples used in this work and their similarities.

Sample	Species	Habit	Collection time	Similarities
S1	*G. carolinianum* L.	Suburb of Nanjing	April, 2011	0.993
S2	*G. carolinianum* L.	Suburb of Nanjing	April, 2011	0.997
S3	*G. carolinianum* L.	Suburb of Nanjing	May, 2011	0.998
S4	*G. carolinianum* L.	Suburb of Nanjing	May, 2011	0.993
S5	*G. carolinianum* L.	Suburb of Nanjing	May, 2011	0.999
S6	*G. carolinianum* L.	Suburb of Nanjing	May, 2011	0.997
S7	*G. carolinianum* L.	Suburb of Nanjing	May, 2011	0.997
S8	*G. carolinianum* L.	Suburb of Nanjing	May, 2011	0.997
S9	*G. carolinianum* L.	Suburb of Nanjing	May, 2011	0.996
S10	*G. carolinianum* L.	Suburb of Nanjing	May, 2011	0.995

**Table 4:** Contents (mg/g) of five selected constituents (compounds **2**, **10**, **11**, **15**, and **16**) in *G. carolinianum* L.

Sample	2 (a)	10 (b)	11 (c)	15 (d)	16 (e)	Total
S1	7.48	41.37	1.80	3.16	4.29	58.10
S2	9.31	49.92	1.79	5.15	5.67	71.84
S3	7.51	41.53	1.81	3.17	4.30	58.32
S4	7.99	32.31	0.96	2.83	2.03	46.12
S5	8.45	43.74	1.48	5.16	3.00	61.83
S6	7.02	34.48	1.08	3.64	2.37	48.59
S7	6.74	30.85	0.99	3.51	2.19	44.28
S8	6.79	36.19	1.37	3.47	2.54	50.36
S9	9.15	43.15	1.49	5.14	3.27	62.20
S10	7.71	37.42	1.58	4.55	2.59	53.85

(a) Gallic acid;

(b) Corilagin;

(c) Methyl brevifolincarboxylate;

(d) Ellagic acid;

(e) Rutin.
